# Exploring the conformational diversity of proteins

**DOI:** 10.7554/eLife.78549

**Published:** 2022-04-21

**Authors:** Avner Schlessinger, Massimiliano Bonomi

**Affiliations:** 1 Department of Pharmacological Sciences, Icahn School of Medicine at Mount Sinai New York United States; 2 Department of Structural Biology and Chemistry, Institut Pasteur, Université Paris Cité Paris France

**Keywords:** protein structure prediction, transporters, G-protein coupled receptors, conformational dynamics, transmembrane protein, artificial intelligence, machine learning, None

## Abstract

An artificial intelligence-based method can predict distinct conformational states of membrane transporters and receptors.

**Related research article** Del Alamo D, Sala D, Mchaourab HS, Meiler J. 2022. Sampling alternative conformational states of transporters and receptors with AlphaFold2. *eLife* **11**:e75751. doi: 10.7554/eLife.75751.

The human body contains a vast number of different proteins that carry out distinct roles. Proteins are made up of combinations of 20 amino acids, each with different physicochemical properties. The number and sequence of amino acids in a protein determine how it will fold into the specific three-dimensional structure or shape that the protein needs to perform its role.

Several proteins, including membrane proteins, do not simply fold into a single conformation. Instead, they need to be able to ‘flip’ between different conformations to do their job. Innovations in the experimental techniques used to determine protein structures – such as cryo-electron microscopy, nuclear magnetic resonance spectroscopy or X-ray crystallography –have provided valuable insights into the different conformations of many membrane proteins. However, these methods are costly and time consuming.

Using computational methods to predict the structures of proteins could allow scientists to fill the gap between protein sequence and structural knowledge, without having to rely on expensive experimental methods (Baker and Sali, 2001). Recently, an artificial intelligence-based method to predict protein structures, called AlphaFold2 (AF2), has taken structural biology by storm (Jumper et al., 2021).

AF2 emerged as a valuable tool for predicting the structures of proteins from their sequences with an accuracy comparable to that obtained by experimental techniques at a fraction of their time and costs, as shown for various biological problems (Evans et al., 2021; Mosalaganti et al., 2021; Tunyasuvunakool et al., 2021; McCoy et al., 2022). Now, in eLife, Diego del Alamo, Davide Sala, Hassane Mchaourab and Jens Meiler report how AF2 can also predict different conformations of membrane proteins (Del Alamo et al., 2022).

To do so, the researchers – based at Vanderbilt University and Leipzig University – used a set of eight membrane proteins representing different structural classes and mechanisms of action. This included five unique transporters (LAT1, ZnT8, MCT1, STP10, and ASCT2), whose structures had been previously experimentally determined in both inward- and outward-facing conformations (Figure 1), and three representative G-protein-coupled receptors (CGRPR, PTH1R, and FZD7), whose structures had been solved experimentally in active and inactive states. None of these proteins were part of the original AF2 training set, which included structures located in the Protein Data Bank (PDB).

**Figure 1.**
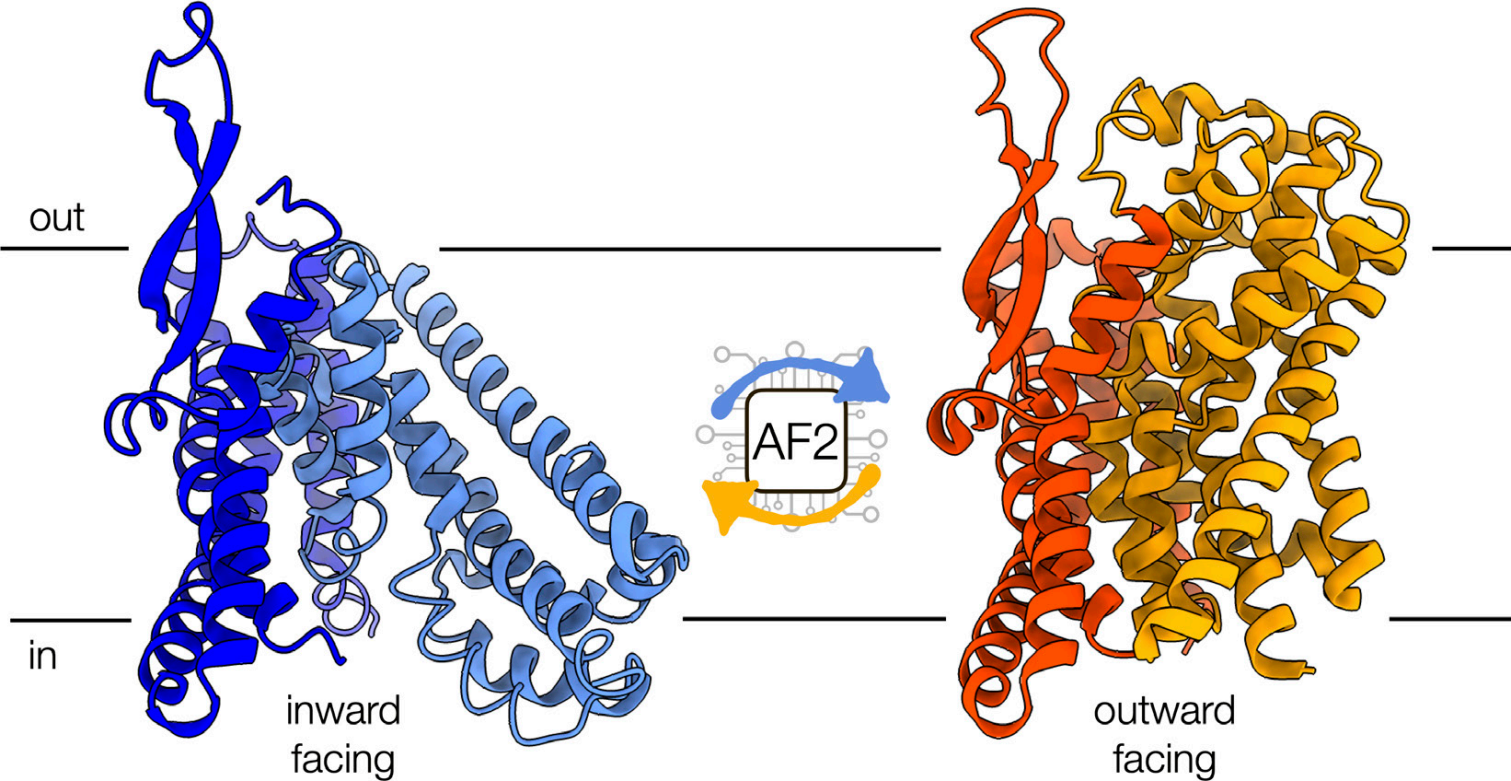
Conformational changes of the alanine-serine-cysteine transporter 2 (ASCT2). An artificial intelligence-based programme, called AF2, can predict the conformational diversity of membrane proteins, such as ASCT2, by modifying the depth of the input multiple sequence alignment. Shown are the cryo-electron microscopy structures of ASCT2 in conformations facing inside (blue) and outside of the cell (yellow). ASCT2 uses an elevator-type alternating access mechanism to transport molecules, which involves a change in the relative orientation of the scaffold (dark tones) and transport domains (light tones) of the protein.

Image credit: inward-facing structure, PDB 6RVX (Garaeva et al., 2019); outward-facing structure, PDB 7BCQ (Garibsingh et al., 2021) (CC BY 4.0).

The researchers then tested the ability of AF2 to model distinct conformational states by varying different parameters of the predictor, such as the number of models generated, and by using known structures of protein homologues as templates. One key feature of AF2 is its ability to generate a multiple sequence alignment (MSA) of evolutionarily related sequences, which is critical for accurate modeling. These MSAs, which can include thousands of sequences, are used by AF2 to identify residues that have co-evolved, thereby highlighting the contacts critical for defining the three-dimensional fold of the protein.

Remarkably, del Alamo et al. demonstrated that by reducing the size of the input MSA or alignment depth from 5,120 to as few as 16 sequences, the conformational diversity explored by AF2 increased, thereby capturing the structures that were experimentally determined in different conformations. This procedure also generated misfolded or outlier models, which were identified by the lack of structural similarity to other models and excluded from further analysis. This provides an important step to distinguish structural models that represent biologically relevant states. Moreover, in some cases using templates as input increased the conformational diversity of the generated models when MSAs with reduced number of aligned sequences (shallow MSAs) were used. Taken together, these results suggest that minor modifications to the input parameters allow AF2 to explore a larger area of the conformational space of proteins to capture distinct, biologically relevant states.

However, the analysis was performed on a relatively small benchmark set of proteins, due to the limited number of membrane protein structures not included in the AF2 training set and resolved in multiple states. Furthermore, del Alamo et al. did not identify a one-size-fits-all protocol that could accurately model the conformational diversity of all the membrane proteins in their benchmark set. A more generalized approach would be useful to study a larger variety of proteins that adopt different conformations, including enzymes and transcription factors. Finally, given the significant role of membrane proteins as drug targets, it will be crucial to assess whether the models generated with the proposed approach can be used for rational drug design, which typically requires accurate modeling of the protein’s amino acid sidechains.

In conclusion, the work by del Alamo et al. extends the scope of AF2 beyond structure prediction of a single state to the exploration of the conformational diversity of proteins. Even though determining the populations of alternative conformations and the interconversion pathways between them still appears to be out of reach, this work represents a crucial step towards describing the dynamic nature of proteins with modern artificial intelligence-based structure predictors.
